# MBPD: A multiple bacterial pathogen detection pipeline for One Health practices

**DOI:** 10.1002/imt2.82

**Published:** 2023-01-31

**Authors:** Xinrun Yang, Gaofei Jiang, Yaozhong Zhang, Ningqi Wang, Yuling Zhang, Xiaofang Wang, Fang‐Jie Zhao, Yangchun Xu, Qirong Shen, Zhong Wei

**Affiliations:** ^1^ Laboratory of Bio‐Interactions and Crop Health, Jiangsu Provincial Key Laboratory for Organic Solid Waste Utilization, Joint International Research Laboratory of Soil Health, Jiangsu Collaborative Innovation Center for Solid Organic Waste Resource Utilization, National Engineering Research Center for Organic‐Based Fertilizers, College of Resources and Environmental Sciences Nanjing Agricultural University Nanjing China

**Keywords:** bacterial pathogen detection, 16S rRNA gene sequencing, One Health

## Abstract

Bacterial pathogens are one of the major threats to biosafety and environmental health, and advanced assessment is a prerequisite to combating bacterial pathogens. Currently, 16S rRNA gene sequencing is efficient in the open‐view detection of bacterial pathogens. However, the taxonomic resolution and applicability of this method are limited by the domain‐specific pathogen database, taxonomic profiling method, and sequencing target of 16S variable regions. Here, we present a pipeline of multiple bacterial pathogen detection (MBPD) to identify the animal, plant, and zoonotic pathogens. MBPD is based on a large, curated database of the full‐length 16S genes of 1986 reported bacterial pathogen species covering 72,685 sequences. In silico comparison allowed MBPD to provide the appropriate similarity threshold for both full‐length and variable‐region sequencing platforms, while the subregion of V3−V4 (mean: 88.37%, accuracy rate compared to V1−V9) outperformed other variable regions in pathogen identification compared to full‐length sequencing. Benchmarking on real data sets suggested the superiority of MBPD in a broader range of pathogen detections compared with other methods, including 16SPIP and MIP. Beyond detecting the known causal agent of animal, human, and plant diseases, MBPD is capable of identifying cocontaminating pathogens from biological and environmental samples. Overall, we provide a MBPD pipeline for agricultural, veterinary, medical, and environmental monitoring to achieve One Health.

## Highlights

- Multiple bacterial pathogen detection (MBPD) is a 16S‐based pipeline for detecting animal, plant, and zoonotic bacterial pathogens.
- The curated reference database of MBPD contains 72,685 sequences from 1986 known pathogen species.
- MBPD provides appropriate thresholds for taxonomic profiling of common 16S variable regions.
- MBPD outperforms other methods in detecting both causal and coinfecting pathogens.

## Graphical abstract

Multiple bacterial pathogen detection (MBPD) provides the accurate and comprehensive detection of bacterial pathogens from biological and environmental samples based on 16S rRNA gene sequencing. By constructing a relatively complete database of 72,685 sequences for wide identification across a broad range of bacterial pathogens causing animal, plant, and zoonotic diseases, MBPD can provide the appropriate threshold for common variable regions of 16S rRNA gene in taxonomy alignment based on the recommendation of in silico experiment. MBPD is freely available at https://github.com/LorMeBioAI/MBPDLorMeBioAI/MBPD.

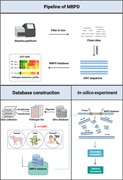

## INTRODUCTION

Pathogenic infections are a formidable challenge in One Health, which is an approach that recognizes that the health of people is closely connected to the health of animals, plants, and our shared environment [1]. During the past two decades, approximately 15 million global deaths per year have been attributed to infectious diseases [2], and approximately 30% of global food production is lost to plant diseases every year [3]. Today, the serious damage to humans and the global economy caused by COVID‐19, the risk of increased zoonotic host diversity, and the pandemic of plant pathogenic bacteria indicate the importance of whole‐habitat (human‐animal‐plant‐environment) detection of pathogens and surveillance for disease management and ecological health [4–6]. In addition, coinfection is common in both natural and agricultural environments [7]. The main problem is that coinfecting pathogens interact synergistically with each other, with the presence of one enhancing the abundance and/or virulence of the other [8]. However, there is no reliable means of detecting coinfection [9, 10]. Many national/international organizations, such as the National Center for Biotechnology Information (NCBI), World Health Organization, and International Society of Plant Pathology Committee on the Taxonomy of Plant Pathogenic Bacteria (ISPP‐CTPPB), have been gathering information on pathogens, but the information is fragmentary and unable to satisfy the requirement of a holistic view of pathogen infections/contamination in biological and environmental samples for implementation of the One Health approach [11].

Pathogen detection is mainly categorized into culture‐dependent and culture‐independent approaches [12]. The former approaches relying on cultivation and biochemical testing are classic experiments for pathogen detection, which provide critical information, including the ability to satisfy Koch's postulates, drug susceptibility, and biochemical tests, but are highly time‐consuming and limited by the culturability of pathogens [13]. Culture‐independent approaches include PCR‐ and high‐throughput sequencing‐based methods. PCR or probe microarrays reduce time in pathogen identification but are overreliant on the specificity and number of primers and prone to false‐positive rates in pathogen detection [14, 15]. Common high‐throughput sequencing‐based methods involve 16S/ITS/18S/*rpoB*/*cpn60* amplicon sequencing and metagenomic sequencing [16]. Given the reported pathogen sequences, sequencing methods are capable of identifying various pathogens within almost all types of microorganisms in the samples [17]. Compared with culture‐dependent and PCR methods, the main benefit of sequencing methods is being high throughput, but the matching analysis pipelines need to be improved to achieve high sensitivity [18].

16S rRNA gene sequencing is widely used in bacterial pathogen detection in animal, food, water, soil, and plant samples [16, 19–23]. However, the scope and sensitivity of pathogen detection using 16S sequencing are highly dependent on the sequencing methods, hypervariable regions sequenced, pathogen database, and analytic pipelines [9, 18]. Short‐read sequencing is high‐throughput and cost‐effective but has limitations in read length [18]. Long‐read sequencing is able to generate long reads for high‐resolution taxonomy but has a limitation of sequencing error rates of approximately 13% [17]. Additionally, the composition and abundance of pathogens can be affected by the use of different bioinformatics tools [12]. For example, the common approaches to data processing include operational taxonomic unit (OTU) clustering or amplicon sequence variant (ASV) approaches [24]. Compared with the OTU‐based approach, the ASV‐based approach has the advantages of fewer sequencing errors and more accurate taxonomic resolution [25, 26]. Moreover, the outcome of pathogen detection fluctuates depending on the reference database, sequencing region, and similarity threshold [27, 28]. Several pipelines for pathogen identification have been developed based on 16S rRNA amplicon data, such as 16SPIP [29], MIP [30], and Krishna [31], but they only target pathogens in relation to human infection or water quality [29–31]. The widespread distribution and transmission of pathogens among multiple hosts and environments require a tool for systematic and rapid detection of pathogens suitable for all habitats to provide a basis for One Health diagnosis and therapy of multiple pathogens that coexist in humans, animals, and plants [1, 32].

In this study, we developed a bacterial pathogen detection pipeline, multiple bacterial pathogen detection (MBPD), to accurately detect pathogens based on 16S rRNA gene sequencing. To comprehensively identify pathogens in samples and meet the demand for accurate pathogen identification under different variable regions in 16S rRNA sequencing, we constructed a curated full‐length 16S database matching the DADA2 pipeline [25] of the ASV‐based approach for data processing and evaluated the optimal similarity based on in silico experiments. MBPD is able to detect a broad range of animal, plant, and zoonotic pathogens, which is essential for food safety, epidemic prevention, disease diagnosis, and environmental monitoring. MBPD is publicly available at https://github.com/LorMeBioAI/MBPD.

## RESULTS

### Overview of MBPD

**Figure 1:**
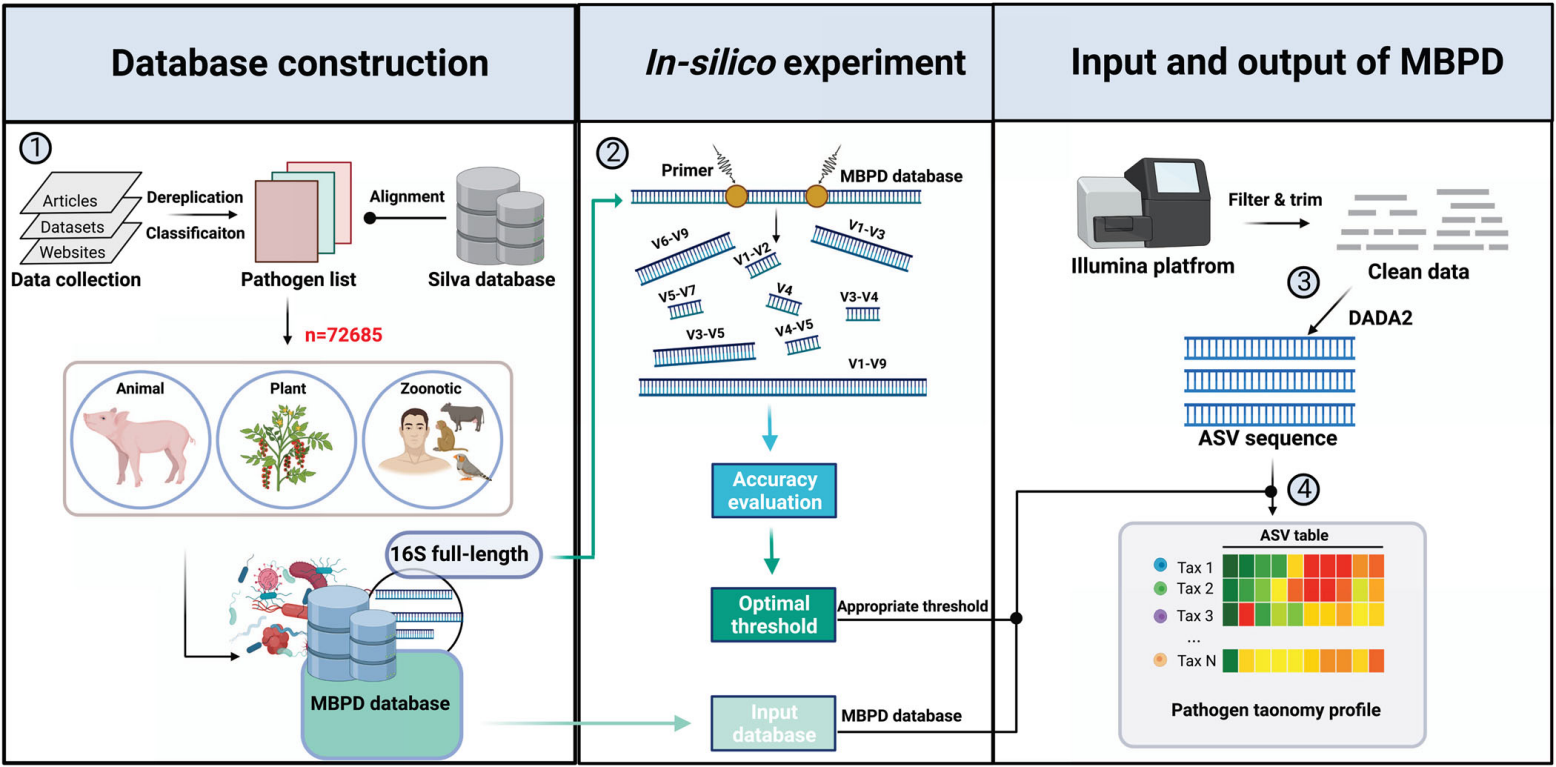
MBPD workflow for open‐view bacterial pathogen detection based on 16S rRNA‐encoding gene sequencing. Bacterial pathogens causing animal, plant, and zoonotic diseases were first collected and curated from publicly available literature, databases, and web resources to construct the multiple bacterial pathogen database (MBPD) with a total of 72,685 full‐length sequences of 16S. Then, the accuracy and appropriate threshold of each variable region of 16S were evaluated through an in silico experiment. The clean data from 16S sequencing of bio/eco samples were subjected to the DADA2 pipeline using the MBPD database to obtain the amplicon sequence variant (ASV) sequences. The feature table of the ASV sequences was assigned using the UCLUST algorithm with an in silico experiment to optimize the cutoffs of similarity for the different variable regions of 16S. MBPD, multiple bacterial pathogen detection.

We developed the MBPD approach for holistic pathogen detection based on 16S sequencing. The input of MBPD is 16S rRNA amplicon data without primers and barcodes, and the output is an ASV table with pathogen taxonomy. The pipeline consists of the following steps: (1) building the MBPD database based on the alignment between collected pathogen information and the Silva 16S database; (2) use of the MBPD database in in silico and sequence‐based experiments to explore the optimal similarity and the accuracy of species/strain annotation using the UCLUST algorithm for the different variable regions of 16S; (3) use of the DADA2 tool for the conversion from 16S rRNA amplicon data to ASV sequences; (4) alignment of ASV sequences with the MBPD database using an appropriate similarity threshold based on various sequencing regions, where 90% is recommended for the V4 and V1−V2 regions, and 80% is recommended for other variable regions (Figure 1).

### A large curated pathogen reference database of 72,685 16S sequences for MBPD

**Figure 2:**
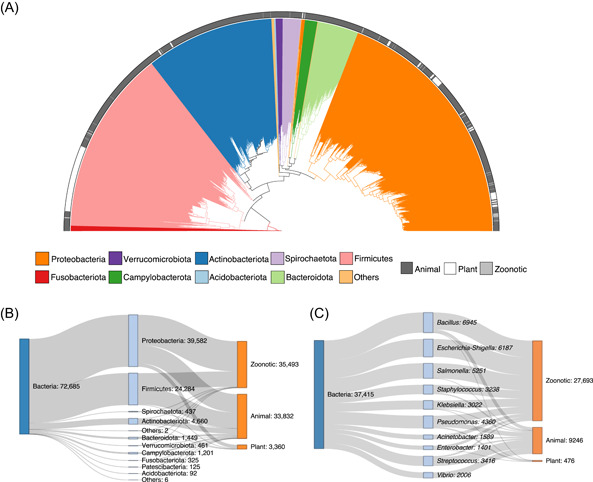
The curated bacterial pathogen database of MBPD. (A) Phylogenetic tree based on the 16S rRNA gene sequences of 1986 reference pathogens. Colors of branches represent the corresponding phyla, and the outer ring denotes animal, plant, and zoonotic types of pathogens. (B) Basic taxonomic composition of the MBPD database at the phylum level. Only the top 10 phyla are shown in the figure, and the remaining phyla are merged as others. (C) Basic taxonomic composition of the MBPD database at the genus level. Only the top 10 genera are shown in the figure.

To construct a comprehensive database of bacterial pathogens causing animal, plant, and zoonotic diseases, we collected a total of 1986 pathogen species from public agencies, databases, and papers (Supporting Information: Tables S1 and S2). In general, they mainly belonged to four phyla, namely, Proteobacteria (758), Firmicutes (558), Actinobacteria (388), and Bacteroidetes (123) (Figure 2A). Then, we extracted sequences corresponding to those pathogens at the species or strain level from the Silva database, called the MBPD database, to improve data compatibility and better identify pathogen diversity. Briefly, the MBPD database is primarily composed of Proteobacteria (39,582), Firmicutes (24,284), Actinobacteria (4660), and Bacteroidetes (1449) at the phylum level (Figure 2B) and *Bacillus* (6945), *Escherichia‐Shigella* (6187), *Salmonella* (5251), and *Pseudomonas* (4360) at the genus level (Figure 2C). In addition, a high number of these pathogens originated from the “zoonotic” group (35,493 out of 72,685, 48.9%) and “animal” group (33,832 out of 72,685, 46.5%) compared to the “plant” group (3360 out of 72,685, 4.6%) (Figure 2B and Supporting Information: Figure S4). Details of the sequence number in each species are presented in Supporting Information: Table S5.

### In silico experiments under different thresholds for sequencing platforms and amplicon targets

**Figure 3:**
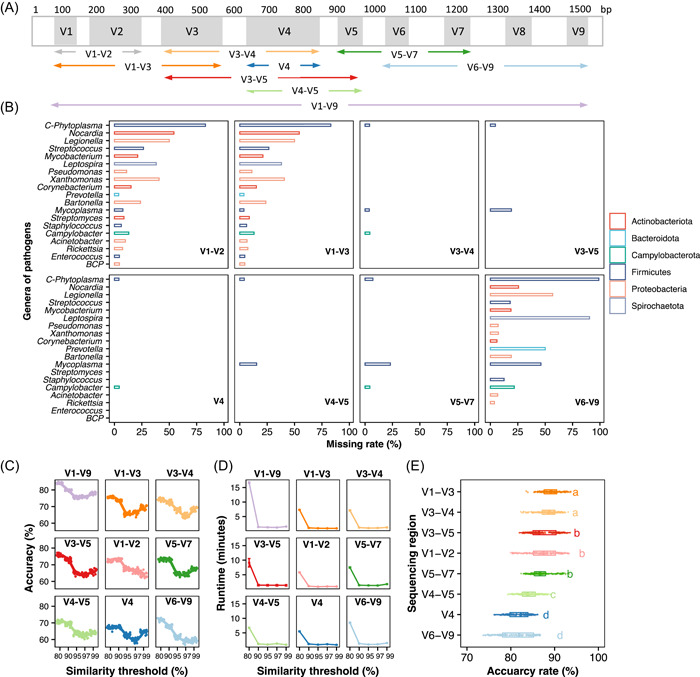
In silico comparison of 16S rRNA variable regions. (A) Common sequencing target of variable regions in Illumina and full‐length sequencing. The 16S full‐length database of MBPD was trimmed and generated in silico amplicons for different subregions based on the location of PCR primers commonly used in microbiome studies. Then, 10,000 sequences were extracted and repeated 30 times for pathogen alignment. (B) Missing rate of pathogens across bacterial genera under in silico experiments. Various facets denote the sequencing target of 16S variable regions (V). Colors denote the missing pathogen at the phylum level. *C‐Phytoplasma* and *BCP* refer to *Candidatus Phytoplasma* and *Burkholderia‐Caballeronia‐Paraburkholderia*, respectively. (C) Accuracy rate of taxonomy assignment for in silico amplicons of the MBPD database with varying similarity thresholds (80%, 90%, 95%, 97%, and 99%) for different variable regions of 16S. (D) Runtime of taxonomy assignment for in silico amplicons with varying similarity thresholds for different variable regions of 16S. (E) Accuracy rate of the subregion in pathogen detection compared to the V1−V9 region. Different lowercase letters indicate significant differences in disease incidence across varieties (HSD post hoc test: *p* < 0.05). HSD, honest significant difference.

The 16S rRNA gene comprises nine variable regions interspersed throughout the highly conserved 16S sequence (Figure 3A). To provide better detection efficiency for various sequencing platforms and amplicon targets, we conducted an in silico experiment to compare the missing rate, accuracy rate, and runtime under different thresholds (Figure 3, Supporting Information: Figures S1 and S2). We found that subregions differed substantially in the extent to which they could match the PCR primer. The V1−V2, V1−V3, and V6−V9 regions performed worst, with 29.3%−33.2% of in silico amplicons unmatched (Supporting Information: Figure S2A), mainly belonging to three phyla (Proteobacteria, Firmicutes, and Actinobacteria) (Figure 3B and Supporting Information: Figure S1). Meanwhile, certain pathogen genera were probably unstably detected by the sequencing of variable regions with a high missing rate by in silico amplicons (Figure 3B). For example, phytopathogenic *Candidatus Phytoplasma* spp. is highly missing in the V1−V2, V1−V3, and V6−V9 regions, while human pathogenic *Mycoplasma* spp. is not well assigned in V3−V5, V4−V5, and V5−V7 (Figure 3B). Next, various sequencing regions showed a distinct discrepancy in the accuracy of pathogen classification (Supporting Information: Figure S2B). The V1−V9 region (full‐length) performed best (analysis of variance [ANOVA]: *F*_8,1341_ = 183.9, *p* < 0.001, Supporting Information: Figure S2B), and the subregions of V1−V3 and V3−V4 were second to V1−V9 after eliminating the difference in the similarity threshold at the sequence level (ANOVA: *F*_7,1192_ = 174.7, *p* < 0.001, Figure 3E). Above all, the choice of similarity threshold substantially affected the accuracy of pathogen identification and runtime. In terms of accuracy, most subregions performed well at the 80% similarity threshold, but the V4 and V1−V2 regions performed best at the 90% threshold (Figure 3C). Although the similarity threshold of 80% is recommended for both full‐length and V3−V4 sequencing approaches to obtain the optimal accuracy of bacterial pathogen detection, it took more processing time at the 80% similarity threshold than at other thresholds (Figure 3D).

### MBPD outperformed other approaches in pathogen detection

**Figure 4:**
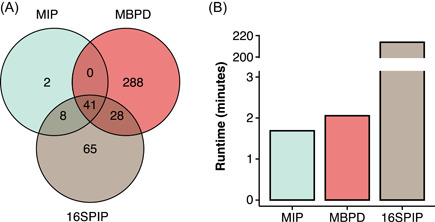
Performance of MBPD, 16SPIP, and MIP in bacterial pathogen detection. (A) Venn diagram displaying the shared and specific species of pathogens detected by MBPD, 16SPIP, and MIP. (B) Comparison of runtime among MBPD, MIP, and 16SPIP. Runtime in min. The colors green, red, and brown denote MIP, MBPD, and 16SPIP, respectively.

We selected 16S data of 50 patient skin samples sourced from Lax et al. [33] to compare MBPD with two published similar pipelines (16SPIP [29] and MIP [30]) using the same data set. For MBPD, we used the similarity threshold of 90% in the process of pathogen alignment based on the results of the in silico experiment (Figure 3C) because they all amplified the V4 region of 16S. In this study, MBPD contained the largest number of identified pathogens among all the pipelines, with the number of detected pathogens reaching 310, and contained most of the other two, especially in MIP (Figure 4A). According to the evaluation of processing time, MBPD is as time‐effective as the MIP pipeline in detecting pathogenic species with the same sample sizes (Figure 4B). Next, we compared the relative abundance of dominant genera (the six most abundant genera) with MIP because 16SPIP can be applied to pathogen identification but not abundance. We identified the dominant genus *Staphylococcus*, which was also detected in MIP, and detected genera not identified in MIP, such as *Corynebacterium* and *Finegoldia* (Supporting Information: Figure S5A).

### MBPD accurately identified the dominant and coinfecting pathogens in real samples

**Figure 5:**
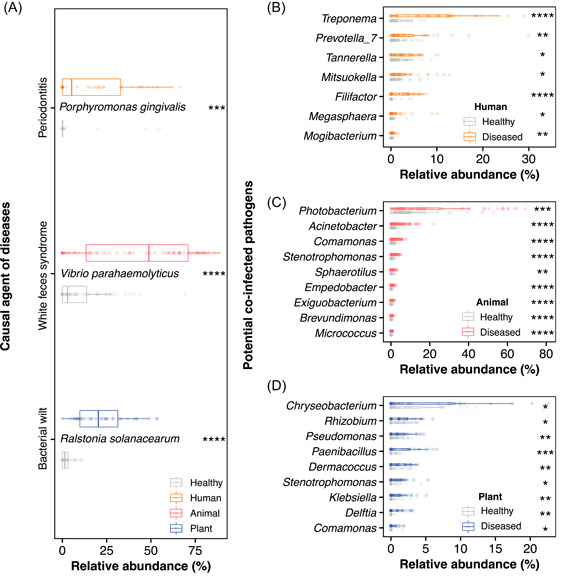
Pathogen detection in the healthy and diseased samples. Differences in the relative abundance of causal agents of diseases in human, animal, and plant rhizosphere samples (A). Relative abundances of other potential genera were enriched in the diseased group between human (B), animal (C), and plant rhizosphere (D) samples. Pairwise Student's *t*‐test was used for statistical analyses (*p < 0.05; **p < 0.01; ***p < 0.001; ****p < 0.0001).

We tested MBPD on paired samples between healthy and diseased individuals for human periodontitis, white shrimp disease, and plant bacterial wilt to further evaluate the performance of MBPD in real environmental samples, which are important sectors of One Health (human, animal, and environment). We used the threshold of 90% for pathogen alignment of all samples because they all amplified the V4 region of the 16S rRNA gene. The abundance of the pathogen community and causal agent of diseases was significantly higher in diseased samples than in healthy samples (Student's *t*‐test, *p* < 0.001, Figure 5A and Supporting Information: Figure S3A−C), but the evenness of the pathogen community in healthy samples was significantly higher than that in diseased samples (Supporting Information: Figure S3D−F). In addition, MBPD was able to identify other potential coinfecting pathogens (Figure 5B−D). For example, the highly enriched genera in diseased samples of humans and animals (*Treponema* and *Photobacterium*; *p* < 0.001 by Student's *t*‐test) could be isolated from the same environment as the causal agent of diseases and seriously threaten the host.

## DISCUSSION

We present MBPD, a method to comprehensively identify bacterial pathogens from bio/eco‐samples using 16S rRNA gene sequencing, focusing on assessing pathogenic contamination in environmental and biological samples. To the best of our knowledge, MBPD represents a One Health‐oriented pathogen detection approach fueled by a large, current reference pathogen database. Key advances underlying our pipeline include (i) a relatively complete database convenient for detection across a broad range of pathogenic bacteria infecting animals and plants and zoonoses; (ii) appropriate thresholds provided for current common 16S amplicon regions for sequence alignment; (iii) high performance in the identification and quantification of pathogens; (iv) accurate identification for both dominant pathogens and their cocontaminating pathogens from biological and environmental samples; and (v) an open‐source tool suitable for constant updating of pathogen databases and processes.

The performance of pathogen identification is affected by the reference database, sequencing region, and similarity threshold [27, 34]. Small databases limit detected pathogen richness [28], and diverse sequencing regions lead to differences in species [27]. Here, we introduced a reference database for pathogen identification based on the Silva database because Silva is comprehensive and updated in a timely manner [35]. However, the optimal similarity threshold under different sequencing regions is still unknown. We conducted an in silico comparison of 16S rRNA gene sequencing under different thresholds [27] and chose UCLUST [36] as the tool for pathogen alignment because UCLUST achieved significantly better performance than all other methods for 16S rRNA gene classifications at the species level [34]. Our assessment showed that 16S full‐length sequencing at the 80% threshold performed best using MBPD, but variable regions will likely reduce the pathogen richness of a microbiome sample. In addition, considering the wider use of the Illumina sequencing platform (lower cost and production of short sequences), we also provided the optimal threshold under various common sequencing regions (V1−V2, V1−V3, V3−V4, V3−V5, V4, V4−V5, V5−V7, and V6−V9) for the best detection results. Next, MBPD enables the detection of cocontaminating pathogens from samples. For example, there are many cross‐infections occurring in a hostile environment [37, 38], but specific pathogens are not clear, or the densities of other pathogens will increase after treatment with the current pathogen. MBPD can also be used for a deeper understanding of pathogens, for example, to elucidate the pathogen diversity in samples and its response to environmental changes.

Although several tools are available to identify pathogens using 16S sequences [29, 30], a benchmark study to assess the accuracy of these pipelines were needed. We downloaded 16S rRNA amplicon‐based sequencing data of 50 patient skin samples from Lax et al. [33] and tested them with these tools. Here, we showed that MBPD might outperform other existing tools when processing the same data: MBPD contains the highest pathogen richness, and the dominant pathogens are consistent with those from other tools, which may result from the more comprehensive database providing a wider detection of pathogens [9]. In addition, we looked at the accuracy of MBPD using 16S rRNA amplicon data of humans, animals, and plants as well as diseased and healthy samples. Each type of sample was adequate for detecting the causal agent of diseases and identifying the potential pathogens.

Although MBPD showed high performance in pathogen detection, there are still limitations and challenges in 16S sequencing diagnosis. First, short‐read sequencing is limited in taxonomical resolution [12, 27, 39, 40]. The performance of the 16S subregion reached a maximum relative accuracy of 90% at the species/strain level compared to 16S full‐length sequencing (Figure 3D). Different strains at the species level or closely related species are occasionally unstably detected using short‐read sequencing [12, 27]. For example, *Staphylococcus* spp. was abundantly recognized as innocuous *S. epidermidis* by MIP but identified as pathogenic *S. aureus* by MBPD (Supporting Information: Figure S5B,C). To date, higher taxonomical resolution in differentiating bacterial strains at the subgenus level has been achieved by long‐read sequencing (PacBio [27] and MinION™ [41]) and protein‐coding taxonomic markers (*rpoB* [42] and *cpn60* [43]), but the target does not perform well in discerning microorganism below the genus level. Second, pathogen identification has primer bias. Studies have indicated that V4 is unstable in identifying enteric pathogens and phytopathogenic *Robbsia* spp. using OTU clustering [40] or the Greengenes database [39]. The current MBPD shows a decent resolution at the species/strain level and accurately detects these two pathogens using the ASV approach with our curated pathogen database (Figure 4A and Supporting Information: Figure S1). This suggested analytical method and database are critical for taxonomic profiling [25, 28]. However, some pathogen species, such as phytopathogenic *Candidatus Phytoplasma* spp., are still difficult to detect (missing rate >75%, Supporting Information: Figure S1) due to the limited strain resolution of 16S for identification [40]. Thus, the identification of *Phytoplasmas* spp. may rely on some protein‐coding taxonomic markers (*rpoB* [42] and *cpn60* [43]) or PCR‐based sequencing approaches applied to multiple genes other than 16S [12]. Finally, sequencing approaches lack direct evidence of lesions. Although the composition and risk of pathogen contamination have been assessed by amplicon and metagenome sequencing approaches [9, 18], the ground truth of causal pathogenic agents, lesions, and disease types require further experimental validations and antilytic method advances. For example, some pipelines, such as MIP, were developed to understand the relationship among pathogens, disease types, and human lesions [30, 44].

## CONCLUSION

We developed a 16S‐based pipeline, MBPD, for the holistic detection of animal, plant, and zoonotic pathogens. MBPD contains the most comprehensive reference database for pathogen identification and quantification with feasible updating. This pipeline outperforms the existing tools for detecting bacterial pathogens and provides the optimal similarity threshold for pathogen alignment from varying biome samples. We anticipate a broad application of MBPD for assessment and diagnosis in the clinic, agriculture, fisheries, and veterinary medicine in the current One Health era.

## METHODS

### MBPD database construction

We first collected information on animal, plant, and zoonotic pathogens from various studies and databases, such as 16SPIP [29], FAPROTAX [45], and Bull *et al*. [46], [47], [48] (for details, see Supporting Information: Table S1). Based on the species level, a pathogenic reference database containing 72,685 sequences was obtained by alignment with the Silva 138.1 SSU Ref NR 99 bacterial database, which was developed with a 99% identity to remove redundant sequences [35], and a label on pathogenic types was added to the basic taxonomy of the Silva database (Supporting Information: Table S4, type). For unclassified pathogens, we further searched for relatively accurate taxonomic information in the NCBI Taxonomy Database (https://www.ncbi.nlm.nih.gov/taxonomy). However, considering that the species and strain levels in the Silva database are not well distinguished [49], we added a label for the corrected species level (Supporting Information: Table S4, species_manual). To construct and explore the phylogenetic relationships of pathogens, the sequences of all species (1986) were aligned using MAFFT [50], and a de novo phylogenetic tree was constructed using FastTree2 based on the maximum‐likelihood method [51]. The phylogenetic tree was further visualized using the iTOL web tool (https://itol.embl.de).

### In silico comparison of 16S rRNA gene sequencing under different thresholds

The in silico analysis was carried out separately using the MBPD database. In silico amplicons demarcating different subregions of the 16S gene were generated by trimming regions defined by established primer sets (Supporting Information: Table S2) using the USEARCH *search_pcr2* command [36]. Sequences were discarded if one or more variable regions could not be matched to a primer pair [27]. To determine the taxonomic resolution afforded by different variable regions and thresholds, 10,000 sequences were randomly selected using SeqKit [52] from each in silico amplicon, and classification was repeated 30 times against the MBPD database using the UCLUST algorithm [36, 53] with a variety of similarity thresholds (80%, 90%, 95%, 97%, and 99%). The accuracy rate, calculated as Accuracy rate = 100 × (accuracy_full‐length_ − accuracy_subregions_)/accuracy_full‐length_, was employed to explore the best performance of various subregions in pathogen identification. The missing rate, calculated as Missing rate = 100 × (total number_phyla_ − miss number_phyla_)/total number_phyla_, was used to reveal the proportion of sequences for each variable region that could not be matched in the in silico experiment.

### Data processing

Sequences with primers and barcodes removed were processed using the DADA2 pipeline [25], which is designed to obtain sequences with a single‐nucleotide difference, known as ASVs. Trimming and filtering were performed on paired reads with a maximum of one expected error per read (maxEE = 1). After merging paired reads and chimera filtering, the phylogenetic affiliation of each 16S rRNA gene sequence was analyzed by the UCLUST algorithm [36, 53] against the MBPD database using the appropriate threshold based on the results of in silico experiments.

### Amplicon sequencing data for testing the performance of MBPD

To compare the performance of MBPD with that of 16SPIP and MIP, we randomly picked 50 patient skin samples from projects [33] targeting the V4 region of 16S for taxonomic profiling, which had been previously tested on the MIP pipeline [30]. In addition, three published V4 regions of 16S data from human, animal, and plant samples, comprising paired healthy versus diseased samples, were downloaded from the NCBI according to accession number: periodontitis (pathogen: *Porphyromonas gingivalis*) [20], white shrimp disease (*Vibrio parahaemolyticus*) [19], and bacterial wilt (*Ralstonia solanacearum*) [54]. The raw sequences were first processed using fastp [55] to truncate low‐quality reads, potential primers, and barcodes. Then, paired‐end sequences were merged using the QIIME *join_paired_ends.py* command [53]. Finally, cleaned fastq files were processed using the MBPD pipeline.

### Statistical analysis

Student's *t*‐tests (two‐sided) were used to test for statistical significance between pairs of samples, where *p* values below 0.05 were considered statistically significant. ANOVA and Tukey's honest significant difference tests was used to determine the statistical significance of multiple comparisons. All statistical analyses were carried out using the R 4.1.2 program (www.r-project.org).

## AUTHOR CONTRIBUTIONS

*Conceptualization*: Xinrun Yang, Gaofei Jiang, Zhong Wei. *Resources*: Gaofei Jiang, Zhong Wei. *Methodology*: Xinrun Yang, Gaofei Jiang, Zhong Wei. *Data curation*: Xinrun Yang, Gaofei Jiang, Yuling Zhang, Ningqi Wang. *Formal analysis*: Xinrun Yang, Gaofei Jiang, Yaozhong Zhang, Ningqi Wang, Yuling Zhang. *Funding acquisition*: Gaofei Jiang, Zhong Wei, Xiaofang Wang, Fang‐Jie Zhao, Yangchun Xu, Qirong Shen. *Investigation*: Xinrun Yang, Gaofei Jiang, Yaozhong Zhang. *Project administration*: Xinrun Yang, Gaofei Jiang, Zhong Wei, Xiaofang Wang, Fang‐Jie Zhao, Yangchun Xu, Qirong Shen. *Supervision*: Xinrun Yang, Gaofei Jiang, Zhong Wei, Yaozhong Zhang. *Software*: Xinrun Yang, Gaofei Jiang, Yaozhong Zhang, Zhong Wei, Yangchun Xu. *Visualization*: Xinrun Yang, Gaofei Jiang, Yaozhong Zhang. *Writing—original draft*: Xinrun Yang. *Writing—review & editing*: Gaofei Jiang, Yaozhong Zhang, Zhong Wei, Fang‐Jie Zhao.

## CONFLICT OF INTEREST

The authors declare no conflict of interest.

## Supporting information

Supporting information.

Supporting information.

## Data Availability

MBPD is publicly available on GitHub (https://github.com/LorMeBioAI/MBPD). Supplementary materials (figures, tables, scripts, graphical abstract, slides, videos, Chinese translated version, and updated materials) may be found in the online DOI or iMeta Science http://www.imeta.science/.
