# Supplementary material for: MBPD: A multiple bacterial pathogen detection pipeline for One Health practices
Source: Imeta. 2023 Jan 31;2(1):e82. doi: 10.1002/imt2.82 (PMC10989770; doi:10.1002/imt2.82)
Supplement: Supplementary file 2 — Supporting information. [file IMT2-2-e82-s002.docx]

# **Supporting Information for**

**MBPD: A multiple bacterial pathogen detection pipeline for One Health practices**

Xinrun Yang^#^, Gaofei Jiang^#,*^, Yaozhong Zhang, Ningqi Wang, Yuling Zhang, Xiaofang Wang, Fang-Jie Zhao, Yangchun Xu and Qirong Shen and Zhong Wei^*^

# **Affiliation**

Jiangsu Provincial Key Laboratory for Organic Solid Waste Utilization, Jiangsu Collaborative Innovation Center for Solid Organic Waste Resource Utilization, National Engineering Research Center for Organic-based Fertilizers, Joint International Research Laboratory of Soil Health, College of Resources and Environmental Sciences, Nanjing Agricultural University, Nanjing, 210095, China

# ***Corresponding Authors**

Email: gjiang@njau.edu.cn (Gaofei Jiang); Telephone number: 025-84396864

Email: weizhong@njau.edu.cn (Zhong Wei); Telephone number: 025-84396864

^#^ Equal contribution to this work.

**This file includes: Figure S1 to S5**

**
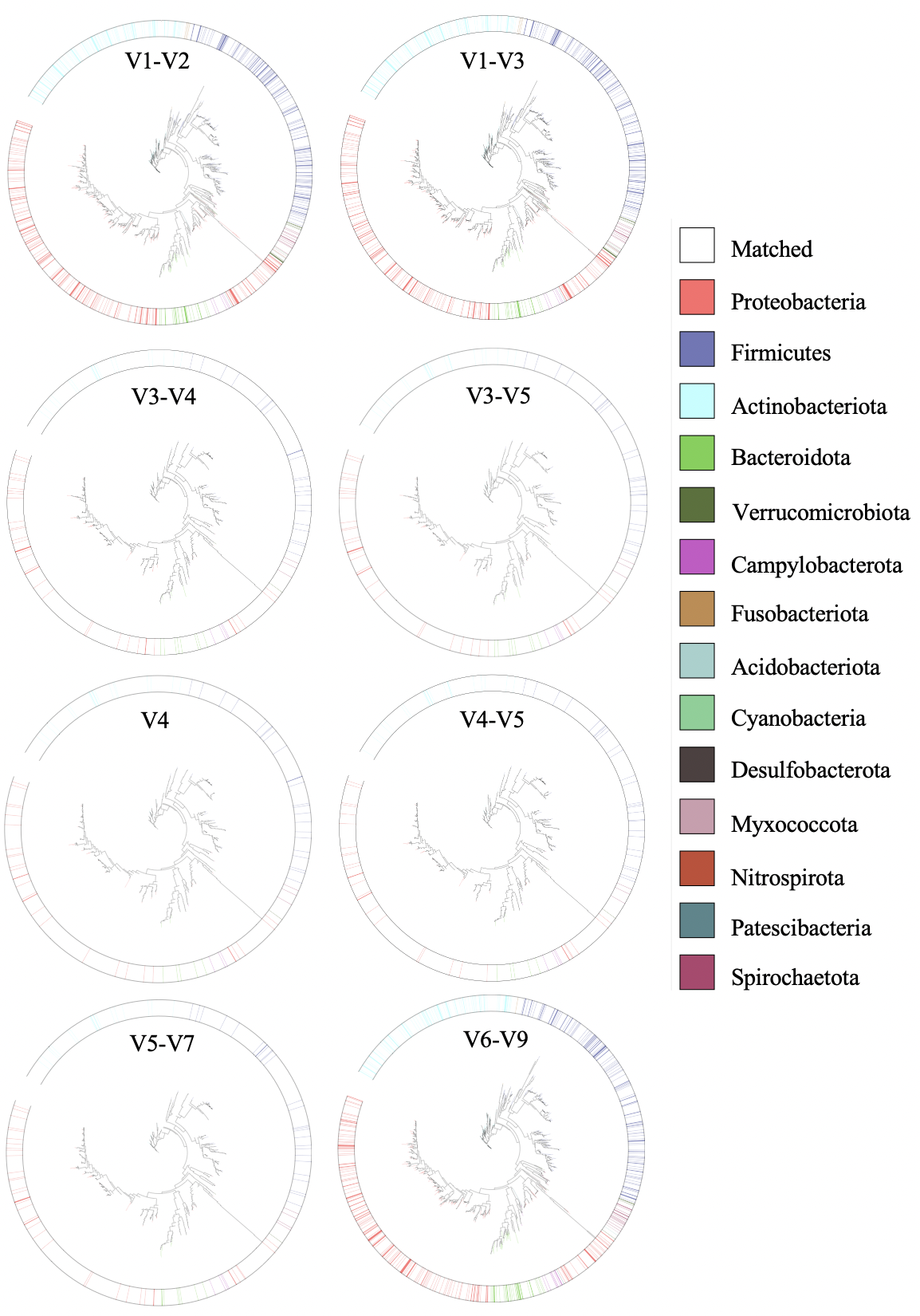
**

**Figure S1 Phylogenetic tree of the 1986 reference pathogen species present in the *in-silico* database.** The color of the outer ring and each branch reflect the phyla of missing sequences in the *in-silico* experiment. Various facets denote the sequencing target of 16S variable regions (V).


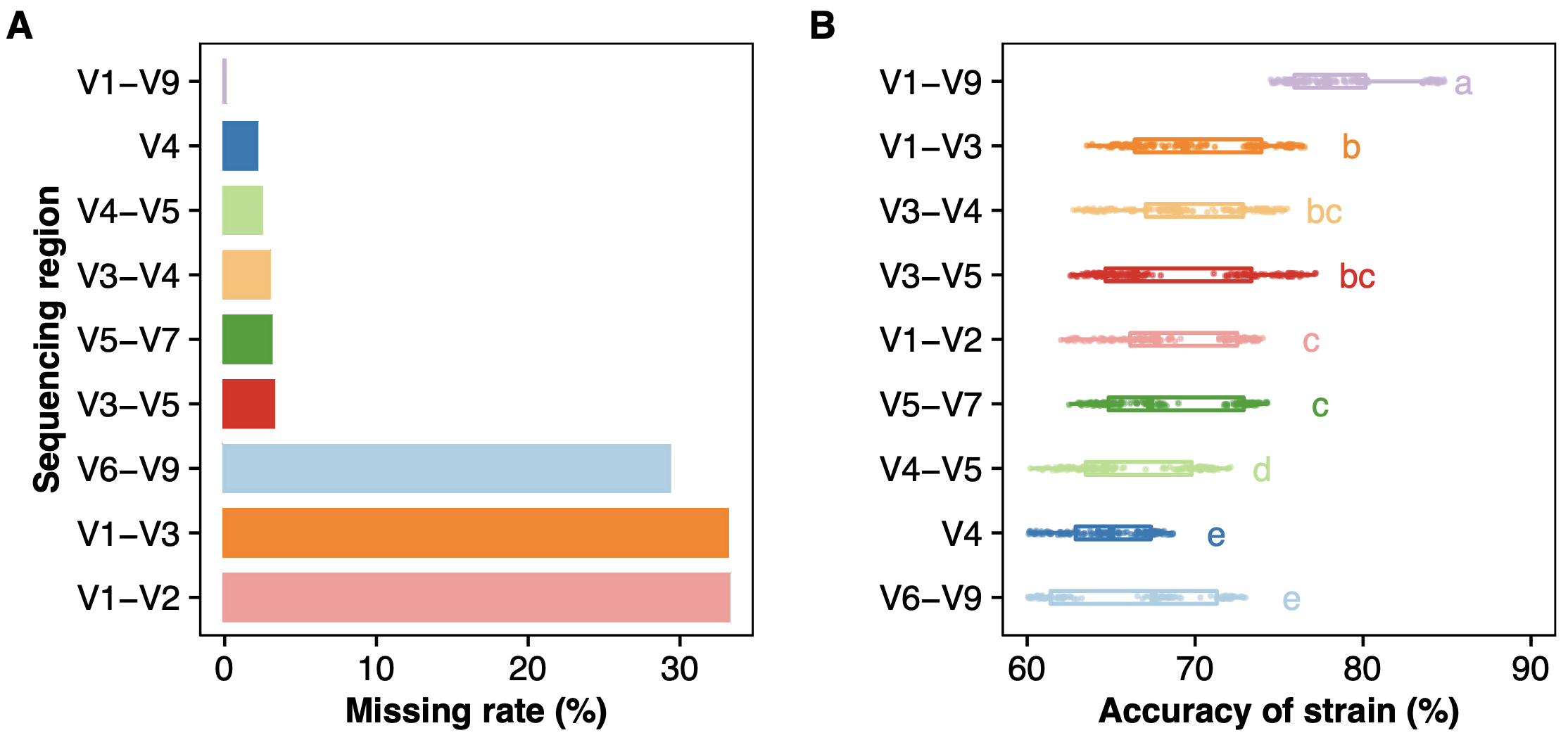


**Figure S2 Benchmarking pathogen sequences with varying sequencing regions and similarity thresholds.** (**A**) The missing rate of sequencing in the *in-silico* experiment. (**B**) Accuracy of *in-silico* amplicons for different variable regions of 16S. V: variable region of 16S.


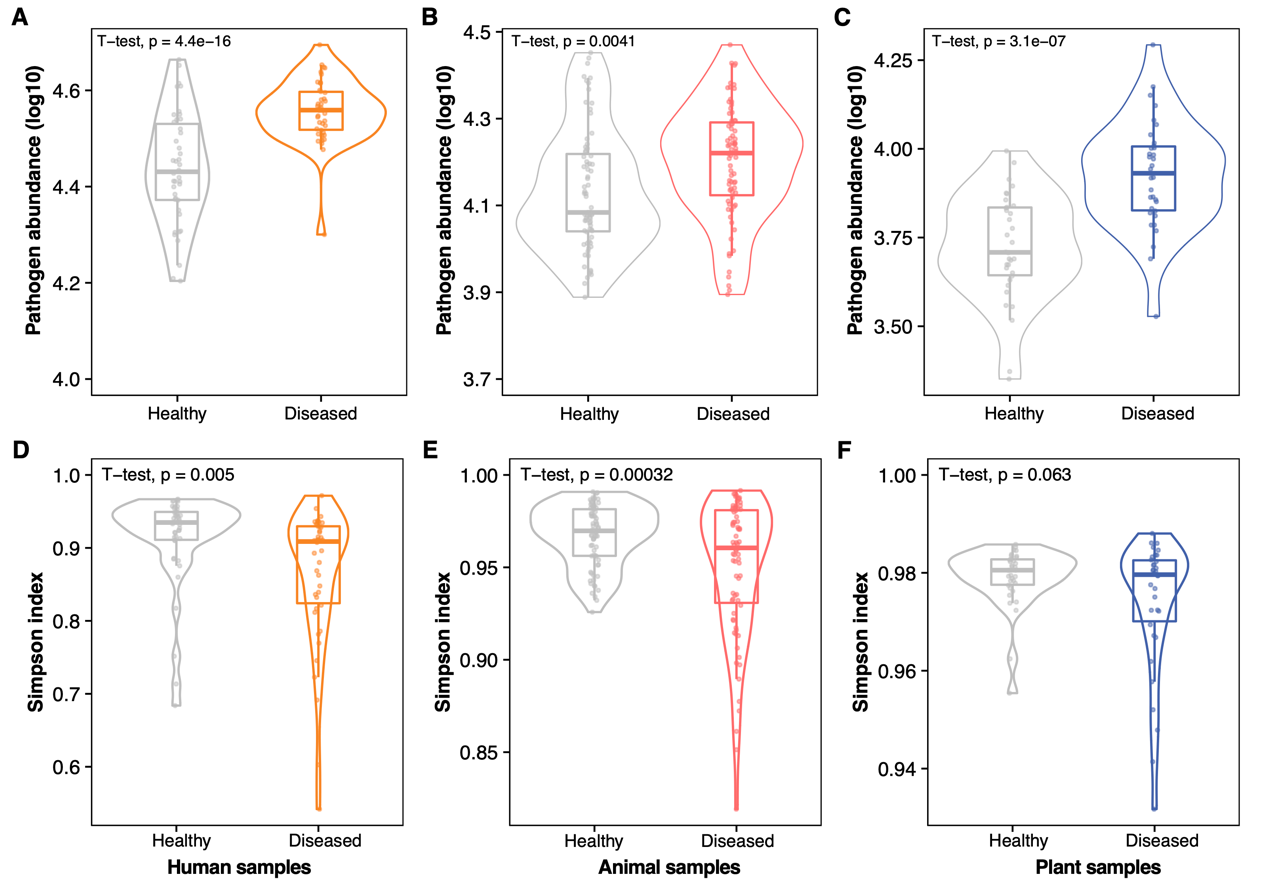


**Figure S3 Comparison of the total pathogen abundances and Simpson index detected by MBPD between healthy and diseased samples in humans (A, D), animals (B, E) and plants (C, F). (A, D)** Healthy and periodontitis disease caused by *Porphyromonas gingivalis* in humans from Chen *et al*., 2018 [1]. **(B, E)** Intestinal samples of white feces syndrome caused by *Vibrio parahaemolyticus* and healthy shrimp from Huang *et al*., 2020 [2]. **(C, F)** Rhizosphere samples of bacterial wilt (Pathogen: *Ralstonia solanacearum*) and healthy tomato plants from Jiang *et al*., 2021 [3] Pairwise Student’s *t* test was used for statistical analyses.


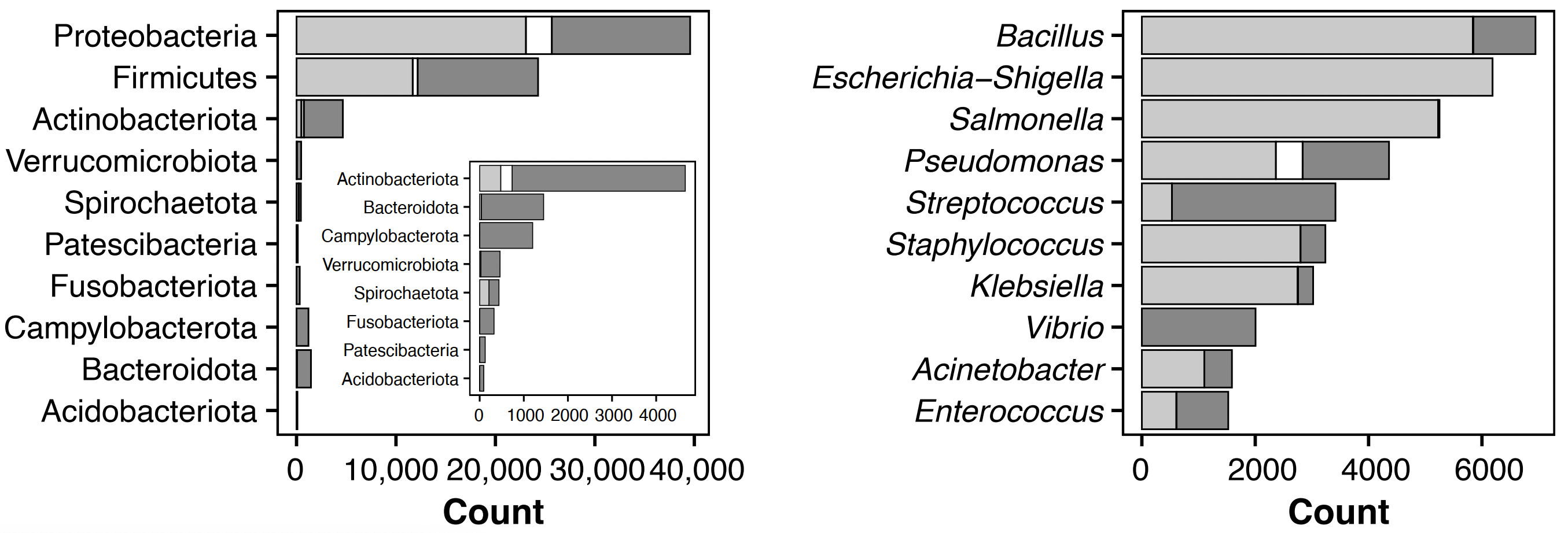


**Figure S4 Basic species composition of the MBPD database.** The top ten phyla (left) and genera (right) of bacterial pathogens in MBPD database are shown in figure.
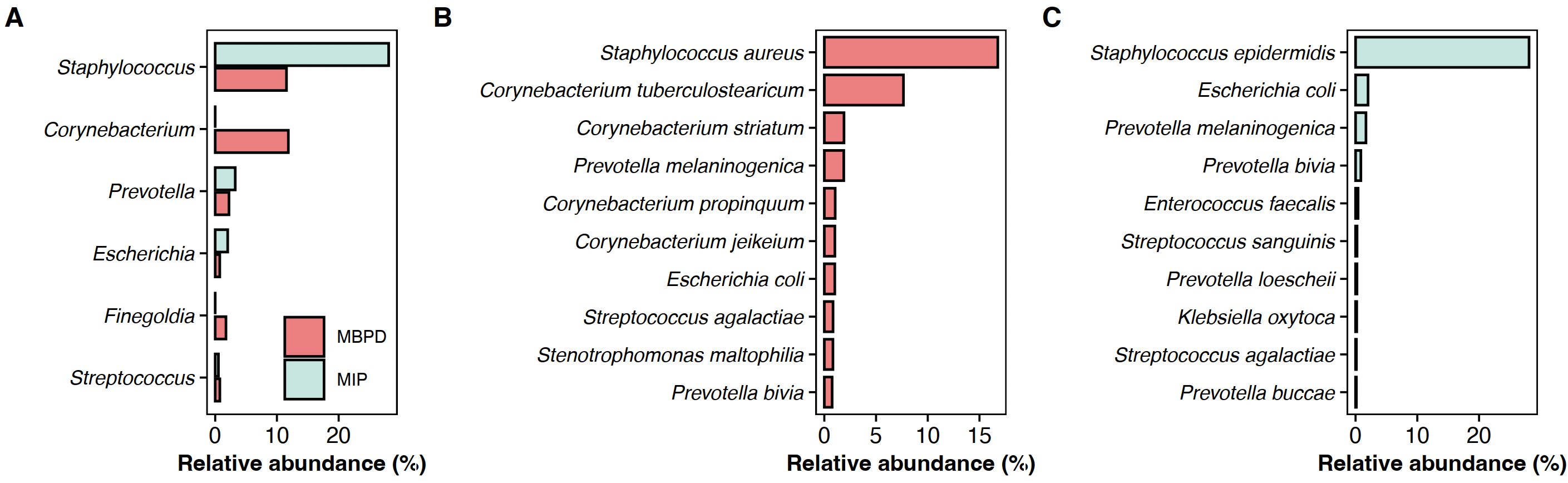


**Figure S5 Differences in the relative abundance of pathogens between MBPD and MIP.** (**A**) Differences in the relative abundance of dominant genus-level pathogens between MBPD and MIP. (**B**) Relative abundance of the top ten species-level pathogens in MBPD. (**C**) Relative abundance of the top ten species-level pathogens in MIP. The colors red and green denote the MBPD and MIP, respectively. MBPD and MIP could accurately identify genus-level pathogens but may be unstable at the species level.

# **Reference**

1. Chen, Casey, Chris Hemme, Joan Beleno, Zhou Jason Shi, Daliang Ning, Yujia Qin, Qichao Tu, et al. 2018. “Oral Microbiota of Periodontal Health and Disease and Their Changes after Nonsurgical Periodontal Therapy.” *The ISME Journal* 12: 1210-1224. <https://doi.org/10.1038/s41396-017-0037-1>

2. Huang, Zhijian, Shenzheng Zeng, Jinbo Xiong, Dongwei Hou, Renjun Zhou, Chengguang Xing, Dongdong Wei, et al. 2020. “Microecological Koch’s Postulates Reveal that Intestinal Microbiota Dysbiosis Contributes to Shrimp White Feces Syndrome.” *Microbiome* 8: 1-13. <https://doi.org/10.1186/s40168-020-00802-3>

3. Jiang, Gaofei, Ningqi Wang, Yaoyu Zhang, Yuling Zhang, Jiabao Yu, Yong Zhang, Zhong Wei, et al. 2021. “The Relative Importance of Soil Moisture in Predicting Bacterial Wilt Disease Occurrence.” *Soil Ecology Letters* 3: 356-366. <https://doi.org/10.1007/s42832-021-0086-2>
